# The experimental study of shunt-decompression arterialized vein flap

**DOI:** 10.1186/s12935-018-0622-z

**Published:** 2018-09-27

**Authors:** Zheng Li, Zhen-wei Zhang, Shao-xiao Yu, Jia-chuan Zhuang, Yu-hai Ke, Yi Xiong, Hui-xin Lin, Wen-feng Chen

**Affiliations:** Department of Hand Surgery, Shenzhen Baoan Shajing People’s Hospital, No. 3 Shajing St. Bao’an District, Shenzhen, 518104 Guangdong People’s Republic of China

**Keywords:** Arterialized vein flaps, Shunt-decompression, Load, Mini pigs

## Abstract

**Background:**

Arterialized vein flap is a kind of unphysiological flap. Unphysiological reconstruction of blood circulation leads to higher load than that supported by physiological flap and is the culprit of flap swelling, blood stasis, skin blistering, and necrosis after flap grafting. To resolve the multiple disadvantages of traditional flap grafting, by introducing the principles of fluid mechanics, shunt-decompression surgery is prepared to decline the circulation preload and improve the prognosis of arterialized vein flap grafting.

**Methods:**

By introducing the principles of fluid mechanics, we established the model of shunt-decompression arterialized vein flap, which satisfied the common properties of general fluid that the interface pressure between object and fluid is reduced when the velocity of fluid is increased and vice versa—the effect of Bernoulli. Under this rule, we anastomose the arterialized vein to the branch of main artery of recipient region or make end-to-side anastomosis, which can maintain the blood flow of main artery, decrease the perfusion of flap, and preserve the decompressive effect of main artery to branches. From March, 2016 to September, 2016, we performed animal experiments on ten male bama mini pigs with average weight of 28 ± 2.35 kg. Superior epigastric artery of pig was used for feeding artery to arterialize the superficial epigastric veins. The total area of flap is 8 cm × 6 cm. End-to-side anastomosis and end-to-end anastomosis were established in experimental group and control group, respectively. Doppler speckle perfusion imaging apparatus was used to monitor the alterations of flap perfusion, blood flow of flap, tissue swelling and survival of flaps.

**Results:**

The average flap perfusion (PU) at 1 week after surgery is 83.62 ± 3.14 in experimental group and 98.14 ± 6.54 in control group, respectively (P < 0.05), indicating the significant reduction of flap blood perfusion in experimental group as compared with control group. As to the survival of flaps, 7 flaps completely survived, 3 showed partial necrosis, and no one was found as complete necrosis in experimental group, while only 3 flaps survived, and 4 flaps and 3 flaps showed partial necrosis and complete necrosis in control group, respectively (P < 0.05).

**Conclusion:**

Based on the physiological features of arterialized vein flap and its problems in clinical application, we improved the anastomosis strategy of flap grafting and obtained excellent experimental outcomes, which provides an insight for the clinical application of arterialized vein flaps.

**Electronic supplementary material:**

The online version of this article (10.1186/s12935-018-0622-z) contains supplementary material, which is available to authorized users.

## Introduction

Vein crisis resulted from regional vein return dysfunction after free flap grafting is the major cause of flap necrosis, especially for arterialized vein flaps [1, 2]. The blood circulation and survival mechanisms of arterialized vein flaps are unphysiological, while it has promising value for clinical application because of its convenience of preparation [3–6]. Unphysiological circulation provide nutrition and blood perfusion for the survival of arterialized vein flaps. The anatomical structure and physiological functions of veins are significantly different from those of arteries. The thinness of vein wall and weakness of vein muscularis make the vein susceptible to the compression of tissue edema [3, 7]. The perfusion of high-pressured and hyperoxia artery blood damages the venous wall, increases tortuosity, narrows the vein diameter, and promotes the formation of thrombin, which dramatically impairs the back-flow of the flap, the balance of flap perfusion and return, as well as its survival [1, 2, 6–12]. By focusing on these characteristics, we proposed that the regulation of blood perfusion load of arterialized vein flap, including reducing the perfusion load and improving the blood return for better perfusion balance, is essentially important [1, 2].

We found in the clinical practice that anastomosis of arterialized vein to the branch of main artery of recipient region or the end-to-side anastomosis can significantly improve the survival of flaps and life quality of the patients.

## Materials and methods

**Fig. 1:**
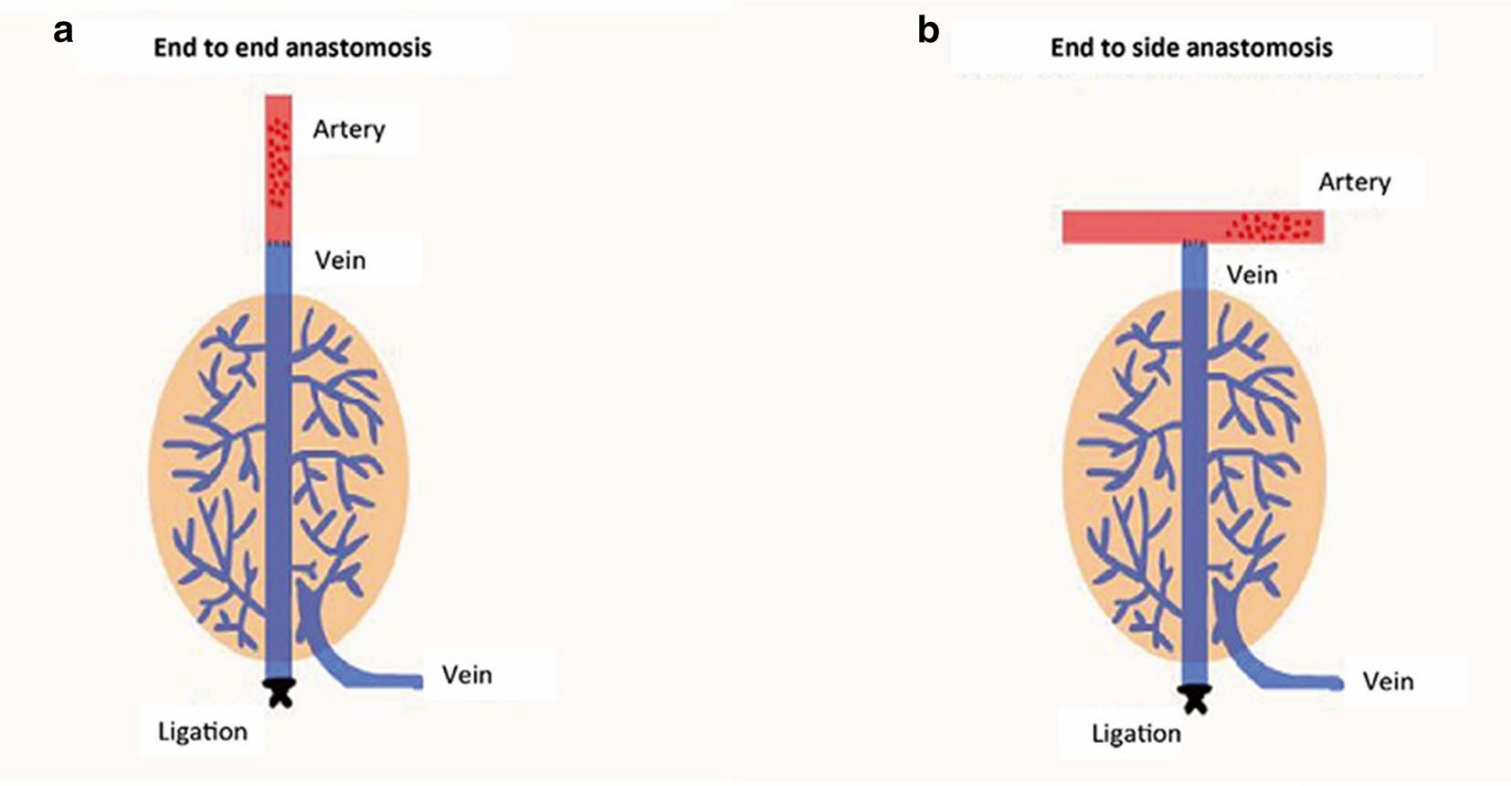
The illustration of end to end anastomosis and end to side anastomosis. End-to-side anastomosis of superior epigastric artery to arterialized superficial epigastric veins was utilized to establish the shunt-decompression mode in experimental group (**b**), while the end-to-end anastomosis of superior epigastric artery to arterialized veins was performed in control group (**a**)

The healthy male bama mini pigs (provided by Laboratory Animal Center, Southern Medical University) were used for our study. We created the animal model and end-to-side anastomosis of superior epigastric artery to arterialized superficial epigastric veins was utilized to establish the shunt regulatory mode in experimental group. The end-to-end anastomosis of superior epigastric artery to arterialized veins was performed in control group (see Fig. 1). Ten bama mini pigs were used and 8.0 cm × 6.0 cm of flaps were prepared respectively on the symmetrical abdominal regions of the pigs. Shunt-decompression surgery was performed in one side of abdominal region as experimental group, while the other symmetrical side was used as control (see Additional file 1: Figure S1). The “three antis” was regularly treated after the surgery. The tensions, colors, swellings, purpuras and filling of vessels were observed every day. The Laser speckle Doppler perfusion imaging apparatus was used to monitor the blood perfusion of the flap. In the late phase, flap tissue necrosis, contractures, and elasticity were indices for investigational observation.

### Statistical analysis

Statistical analyses for flap perfusion parameters were carried out using Prism 6. All the perfusion parameters were expressed with Mean ± SEM. The comparison of flap blood perfusion was performed with unpaired two-tailed t-test. The comparison of perfusion pressure and dynamic blood distribution data was used repeated-measures ANOVA analysis. Flap survival data was analyzed by using χ^2^ test. A P value of less than 0.05 was considered significant.

## Results

**Fig. 2:**
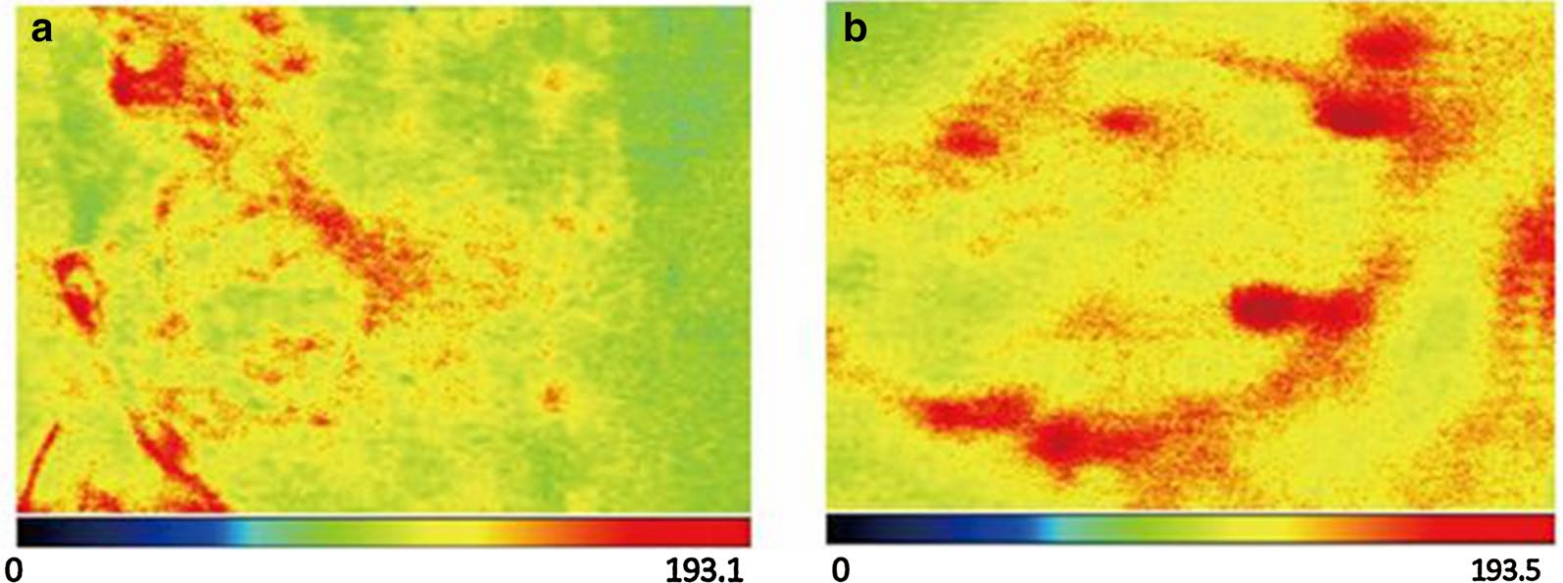
The blood perfusion distribution of flaps in control and experimental group at 1 week after the surgery. **a** The blood perfusion distribution of flap in control group. **b** The blood perfusion distribution of flap in experimental group

**Fig. 3:**
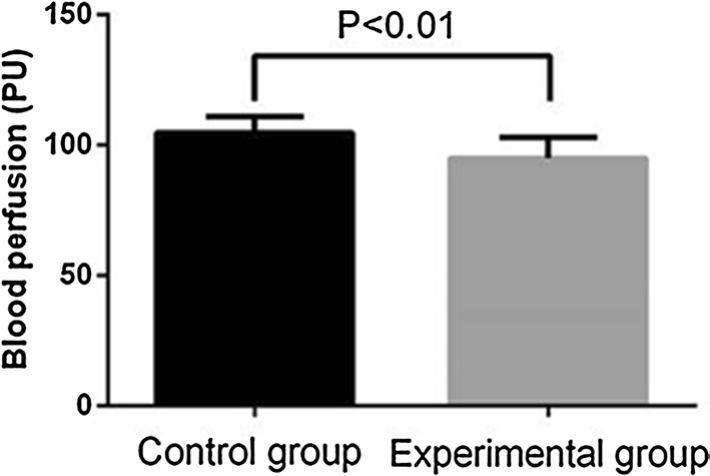
The average perfusion distribution of flap in control and experimental group at 1 week after the surgery. The average blood perfusion is reduced in experimental group as compared with control group

**Fig. 4:**
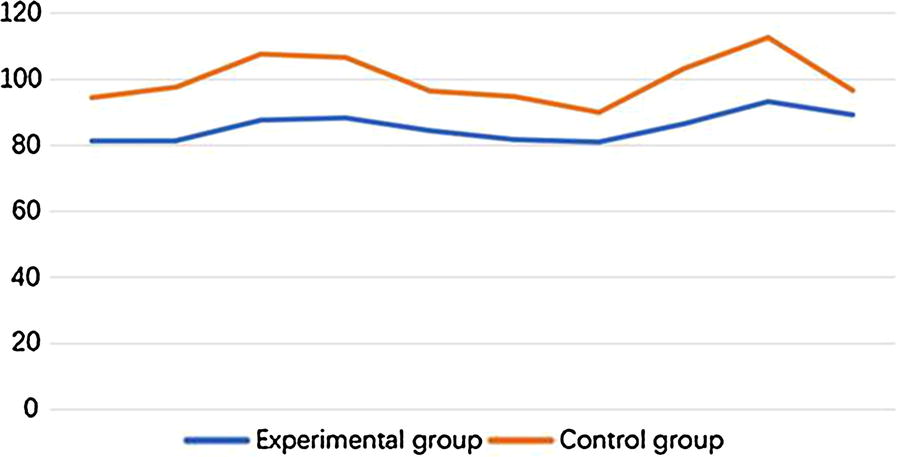
The average blood perfusion pressure of flap in control and experimental group at 1 week after the surgery. The average blood perfusion pressure is reduced in experimental group as compared with control group

**Fig. 5:**
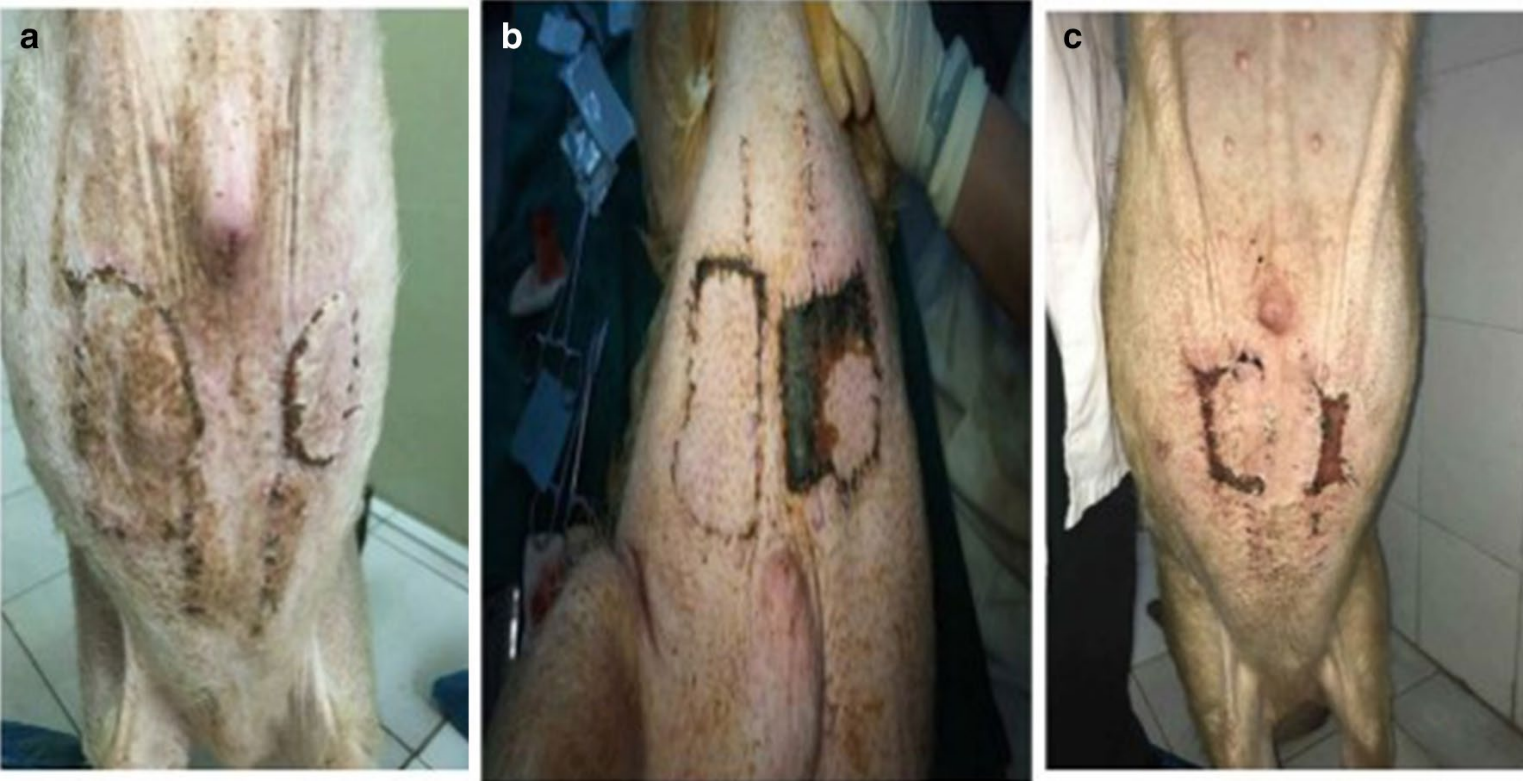
The survival outcomes of flaps in the study. **a** complete survival; **b** partial necrosis; **c** complete necrosis

The average flap blood perfusion (PU) at 1 week after surgery in experimental group is 83.62 ± 3.14, which is significantly reduced as compared with 98.14 ± 6.54 in control group (P < 0.01, see Figs. 2 and 3). As for the blood perfusion pressure, the average perfusion pressure in experimental group is also lower than that of control group (P < 0.0001, see Fig. 4). In the analysis of survival of flaps, 7 flaps completely survived, 3 showed partial necrosis, and no one was found as complete necrosis in experimental group, while only 3 flaps survived in control group, and 4 flaps and 3 flaps showed partial necrosis and complete necrosis, respectively (P < 0.05, see Fig. 5). As to epidermolysis and edema, we did not find any significant difference between the two groups.

## Discussion

Unphysiological arterialized vein flaps has certain clinical applications due to its advantages, such as its easy dissection, free selection of flap region, and no swelling of flap [3–5, 13]. In 1981, Nakayama et al. [14] reported the first successful experimental study of arterialized vein flap grafting, which raised the upsurge of medical studies of vein flaps and paved the way for the clinical application of venous flaps. After 3 decades of development, some clinicians abandoned its use due to its disadvantages, including swellings and congestion of the flaps, and low survival quality [3, 6, 15].

Venous flap is unphysiological and has low survival rate and low quality. As compared with physiological flaps, venous flaps showed more exudation, tissue edema, thickness of dermis collagen fiber and elastic fiber, fiber disarrangement and flap hardness and contraction [3, 6, 9, 15]. These characteristics restricted its application to repair of small wounds, such as skin and soft tissue defect in fingers [6]. Thus, how to improve the survival rate and life quality of the venous flap is a demanding challenge for clinical practice [4, 5].

The characteristics of microcirculation of arterialized vein flaps can be analyzed by using the function: R = 8ηL/πr^4^. This function suggests that blood resistance is inversely correlated with fourth power of radius, indicating small reduction of vein radius may dramatically increase the resistance of veins. When the drive force to propel the blood flow in vein is equal to the resistance, the blood flow will be blocked and vein thrombosis is formed. Besides, the imbalance between blood perfusion and return may play a key role in microcirculation dysfunction in arterialized vein flap grafts [1, 2, 14]. The high blood perfusion of vein may damage the endothelial cell of the vein and lead to the formation of white thrombin [2, 4]. As the thrombin blocked the blood flow of the veins, it will increase the openness of arteriovenous shunt and reduce the exchange of nutrients and wastes in the capillaries and result in subsequent accumulation of acidic substances in the microcirculation [8, 10, 16]. This vicious cycle will finally lead to the microcirculation dysfunction of the flap.

Recently, with the progress of free flap studies, the balance between flap perfusion and return is particularly emphasized. Li et al. proposed that reducing the blood supply of flap can increase the survival rate and quality of the flap, when the diameter of return vein is small or venous return insufficiency occurred. Other researchers also argued that the flap is much more tolerant to low blood perfusion, but less tolerant to venous return insufficiency [1, 2, 7, 9, 10]. If a number of capillaries are opened to maintain 25%–30% oxygen content diffuse into the flap tissue, it is enough to support the survival of flaps. We also found that the balance of blood perfusion and return is directly correlated with the final survival and quality of the flap graft in the animal and clinical studies. Due to the unphysiological style of circulation of arterialized vein flap and the intrinsic properties of vein anatomy, physiological functions, and venous valve, blood is prone to be static in the flap veins and leads to the edema, congestion, blistering and necrosis of the flaps [2, 3, 7–10]. The imbalance of blood perfusion and return may easily exacerbate the dysfunction of flap metabolism, and even results in the failure of microcirculation, which is an important restricted factor for flap survival [2, 7–9, 12, 16]. In the clinical practice, we found that the initial indices of flaps and late survival quality will be significantly improved, even close to the states of survival quality of the physiological flap, when an arterialized vein is anastomosed to a branch of the main artery or end-to-side anastomosis is made. Our current animal study demonstrated above conclusions.

Bama mini pigs were selected for our study because their skin texture and blood circulation system is similar to those of humans [17, 18]. Besides, the convenience of flap dissection, appropriate vessel diameter for end-to-side anastomosis, and easy to observation are also advantages of bama mini pigs for this study [17, 18]. In the experiment, the rules of microvessel operation should be followed due to the high sensitivity of pigs’ vessels. The maintenance of suitable temperature and effective anesthesia are required. The wound should be moist to prevent the dryness of the vessels. What’s more, the branch of free segment of the superior epigastric artery should be ligated to keep the independence of superior epigastric artery as the supply vessel. Finally, to avoid the spasm of vein, any operations should be manipulated lightly and mildly.

We proposed two mechanisms to interpret the improvement of survival quality of arterialized vein flap by using creative shunt-decompression style. Firstly, the anastomosis of arterialized vein to the branch of main artery can decline the preload of flap circulation. Secondly, when the pressure of flap is increased, the blood resistance of the branch will also raise, thus more blood will flow through the other branches of the main artery, which will reduce the blood perfusion of the flap.

## Conclusion

This study demonstrated our shunt-decompression hypothesis that shunt-decompression surgery effectively improved the indices of the arterialized vein flap, including reduced flap swellings, no blisters formed, red skin color, and normal blood flow. In the late phase, flap texture, skin color, elasticity, and contracture are also improved, indicating that its grafting quality is close to physiological flap. Furthermore, epidermolysis and edema were not significantly different between the two groups. We know this surgery may have the disadvantages, for instance, the operative time for the surgery is difficult to control, future studies were required to explore the appropriate time period for the surgery. In all, this study further promoted the advantages of arterialized vein flap and provides more evidence to support the shunt-decompression arterialized vein flap for clinical applications.

## Additional file

**Additional file 1: Figure S1.** The illustration of anastomosis and flap transplantation. A. End-to-end anastomosis during surgery was shown; B. Two symmetrical abdominal regions were utilized as experimental and control group, respectively.
