# Supplementary figures and images for: The experimental study of shunt-decompression arterialized vein flap
Source: Cancer Cell Int. 2018 Sep 27;18:148. doi: 10.1186/s12935-018-0622-z (PMC6161395; doi:10.1186/s12935-018-0622-z)

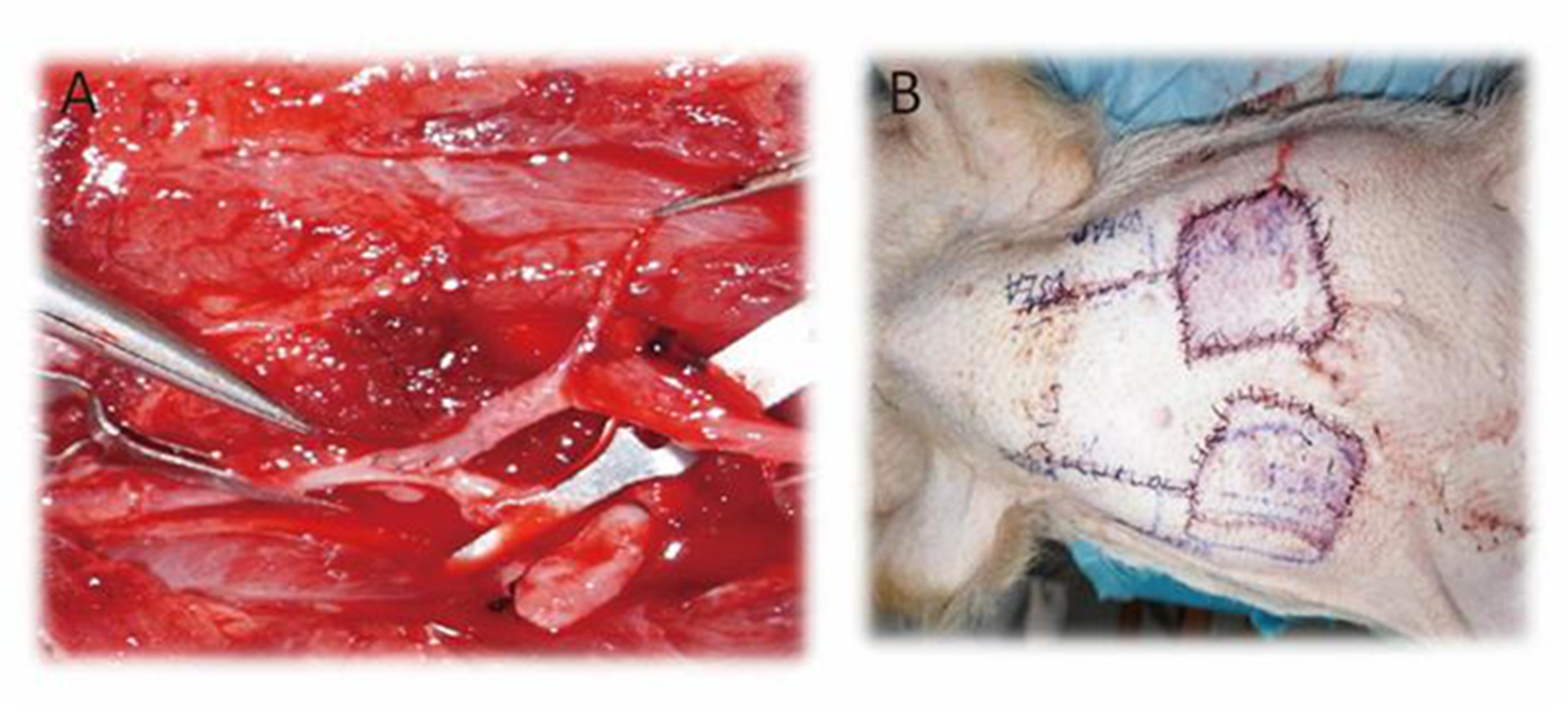

Supplement: Supplementary file 1 — Additional file 1: Figure S1. The illustration of anastomosis and flap transplantation. A. End-to-end anastomosis during surgery was shown; B. Two symmetrical abdominal regions were utilized as experimental and control group, respectively. [file 12935_2018_622_MOESM1_ESM.tif]
